# Comparison of a Bayesian and a regression model for stimulus classification

**DOI:** 10.1186/1471-2202-12-S1-P178

**Published:** 2011-07-18

**Authors:** Lena S Köpcke, Julia Furche, León M Juárez Paz, Thomas Kneib, Jutta Kretzberg

**Affiliations:** 1 Institute of Mathematics, University of Oldenburg, D-26111 Oldenburg, Germany; 2 Institute of Biology and Environmental Sciences, University of Oldenburg, D-26111 Oldenburg, Germany

## 

The central nervous system relies on spikes of retinal ganglion cells (RGC) as the only source of information about the visual environment. Therefore, the RGC response must contain information about e.g. velocity and luminance of an object. A common approach to find out which spike train features encode different stimulus properties is to estimate stimuli by assigning the observed spike trains to stimulus classes according to their features [1]. Here, we compare two statistical methods – a Bayesian and a categorical regression model – for stimulus estimation.

Bayesian stimulus estimation has been used in neuroscientific literature for many years [2]. Its basic idea is to determine the most probable stimulus given the observed response by using prior knowledge about the probability of stimulus occurrence combined with the statistics of response properties, conditioned on the stimulus. The second approach, a regression model based on categorical ordered data, is new to the field of neuronal stimulus estimation. The so called bivariate cumulative probit model [3] assumes that the bivariate ordinal response variable (e.g. the combination of ordered light intensity and velocity) is a discretization of a continuous variable that cannot be observed directly but can be described by a standard linear model with spike train features as covariates.

**Figure 1:**
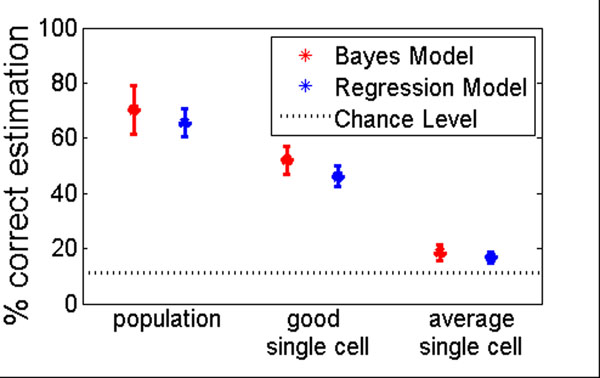
Comparison of the Bayesian and the regression model. Average and standard deviation of the percentage of correct classifications for the cell population and two single cell examples, one with high and one with average classification performance.

We analyzed multi-electrode recordings of carp RGC. The retina was stimulated with a moving pattern of bright dots on a dark background. Stimulus velocity and intensity remained constant for one second and then changed instantaneously in an alternate way. We classified responses to a subset of the stimuli which could be naturally ordered. It comprised the combination of three constant velocities and three light intensity changes, yielding nine stimulus classes. Analyzing the activity of 114 simultaneously recorded RGC responses, latency and spike count were used as input variables for both methods. The performance of each model was measured as the percentage of correct classification averaged over eight trials with 16 stimulus repetitions per trial (Fig.1).

Both approaches led to similar results both for the classification based on the population response combining all 114 cells and for almost all individual cell responses. On average, the Bayesian model yielded slightly higher classification performances than the regression model for the population estimation and most of the single cells. Additionally, less CPU time was needed for the Bayesian analysis. The regression model, however, yielded on average a slightly lower standard deviation of correctly classified spike trains. So both methods proved to be well suited to analyze neural coding.

Comparing both models, the Bayesian approach has the advantage that it does not require a specific order of the response and can include prior knowledge of stimulus occurrence. On the other hand, the cumulative probit model allows for a generalization to higher dimensional responses without much computational effort. It also has the advantage to be intuitively comprehensible and flexible with respect to the number and choice of covariates.
